# Public Schools

**Published:** 1863-03

**Authors:** 


### PUBLIC SCHOOLS.

If any parent will take the trouble to visit any of the Public Schools of New York, say No. 50 in East Twentieth street, for girls, there cannot but be made an impression of admiration of the order, system, promptitude, and efficiency, exhibited in every department. Punctuality, Promptitude, and Truthfulness, are instilled into the minds of the children steadily, and in every possible way. They are taught to think. Their self-respect, their consciences, are constantly appealed to. The punishments are moral, more than physical or corporeal. They are excited, encouraged, or shamed, according to the disposition and temperament. The great results are, accuracy and progress—a progress really astonishing to those who patronize private or more select schools.

We heartily accord with the justness of the sentiments of the intelligent editor of the *Brooklyn Daily Times:*

"We are persuaded that very few of our citizens, even of those who have children in the Public Schools, appreciate the completeness and variety of the instruction given. Many send their children to private schools, because they consider it "respectable" to pay for being exclusive, and different from the majority. For people who cherish this silly, shallow vanity, this preferring of appearance to reality, we have no respect, and do not care to write a line for their perusal. But there are others who, from ignorance of the mode and extent of the studies pursued in the Public Schools, infer that necessarily, because it is paid for, private school tuition must be better and more thorough than that of the Public Schools. We can only advise such to visit the schools (at any of which they will be welcomed by the principal and trustees), and they will soon be convinced of their mistake. We are glad to hear, that not only is the aggregate attendance in the schools increasing, but (as set forth by the City Superintendent in his recent report), the regularity of attendance is augmenting. And not only so, but that the increased attendance, if there be any difference, comes yet more from the children of parents who are able to pay for tuition, than from those who are so circumstanced that public school tuition is for their children the only alternative from no tuition at all.

"These lads (of No. 16, Brooklyn, Dunkley, Principal), who were aged about thirteen or fourteen, went through a variety of studies while we remained in the room. First, the teacher toak up a reader —selected a chapter at random, and set them to parsing. Each boy parsed a word, the next in order the following word, and so on all round and round the class. There was no hesitation, and but a single error, which was instantly detected and rectified by the next lad to the one who made it. In fact, the boys were as attentive as a line of soldiers counting off their numbers, and as prompt and ready in their statements as we should be in repeating the Doxology. Had it not been for the complete acquaintance they evinced with the structure and composition of the sentences, one might almost have been tempted to fancy that it had been a specimen of parsing printed in the book and committed to memory.

Another of the exercises related to philology. The boys were given a word by the teacher, which they wrote on the black-boards, with its affixes, suffixes, prefixes, &c., defining orally the original meaning, and the modifications of the meaning by the changes of form which it might be made to undergo in forming kindred words. Here again, though the words were often recondite, the pupils gave prompt and correct answers. And, from an inspection of some of their written compositions, with which we were favored by the principal, subsequently, it was evident that the lads knew how to apply the knowledge of language thus obtained, to the construction of sentences, and the correct and creditable expression of their sentiments.

"There was also an examination in the rudiments of mathematics, and one in the rules of deliberative assemblies and the Constitution of the United States. These, also, they passed very creditably.

"We were most struck, however, with the proficiency of these boys in arithmetic, both on the slate and mental. The mere effort of memory which they accomplished, in retaining the terms of the questions propounded, was surprising. Each answer was given in the form of a complete problem, logically worked out to its conclusion, through all its intermediate stages of proof. The intricacy of some of the calculations involved, the speed and accuracy with which the solution was given, the completeness and finish with which the whole operation was accomplished, and, above all, the unflagging interest in the lessons which the teacher contrived to sustain in the minds of his pupils, impressed us as the very perfection of successful instruction. After seeing the proficiency of this class in arithmetic and kindred branches, we were not astonished to hear, that no less than sixteen boys have, within a very brief period, gone straight from this class into responsible mercantile employment."

New Yorkers cannot adequately appreciate the obligations under which they are laid to the teachers of the Public Schools, for the fidelity, the assiduity, the patience, self-denial, and painful efforts necessary in bringing children forward in their studies in the manner described. It is a harder work, more trying on mind and body and heart, than plowing corn or chopping wood; and that they richly deserve the compensation they receive, the reader may easily be convinced, by trying to take care of a class of a dozen for half a day.

There are persons in New York, as well as Brooklyn, who do not send their children to the Public Schools, for fear that there is some discredit attached to it. Visions of "contamination" arise before their minds, at the thought of *their* children mixing with "everybody's children." But these same children will have to associate in a variety of ways with "everybody," when they go out into the great world of practical life. Will any parent object to having his child associate with children who are above them, socially? They would rather prefer it, in the hope that their own children would be elevated by the association. Let such, then, have the generosity to do something towards elevating the children of those who are below them; and thus do unto others as they would have others do to them. But it is not likely that those who are so much afraid of their children being "contaminated," have sufficient greatness of soul to help others up. Poor human nature! we are ashamed of some of your phases! A man who is willing to be helped out of the mire, and then is unwilling to help others, is no man at all—he is a thing. It would greatly add to our estimate of the Public School system, if the children were confined four hours, instead of six, a day, and had no lessons to learn out of school; then they might have bodies and constitutions at twenty-one, as well as minds.

